# The Suicide-Risk Factor – Data Query Tool (SURF-DQT): easy and handy access to the exhaustive base of highest evidence suicide risk factors

**DOI:** 10.1265/ehpm.24-00113

**Published:** 2024-12-07

**Authors:** C.-E. Notredame, M. Ford, N. Jabari, O. Bhuiyan, S. Richard-Devantoy

**Affiliations:** 1 CHU Lille, Psychiatry Department, F-59000 Lille, France; 2 PSY Lab, Lille Neuroscience & Cognition Centre, INSERM U1172, Lille University, F-59000 Lille, France; 3 Groupement d’Étude et de Prévention du Suicide, F-86280 Saint-Benoît, France; 4 MAP Centre for Urban Health Solutions, St. Michael’s Hospital, Unity Health Toronto; 5 Agency Undone; 6 McGill Group for Suicide Studies, Department of Psychiatry, McGill University, Montreal (Quebec), Canada; 7 CISSS des Laurentides, St-Jerome (Quebec), Canada; 8 Douglas Mental Health Research Institute, Montréal, Quebec, Canada

**Keywords:** Suicide, Suicide attempt, Suicidal behaviour, Suicidal ideation, Risk factor

## Abstract

The extensive number of publications on suicide risk factors presents a practical challenge for clinicians, policy makers, and researchers to translate the research findings from academia to the improvement of individual suicide predictions and collective prevention. The Suicide Risk-Factor – Data Query Tool (SURF-DTQ) is a web application that collates organized data and helps with meta-analysis of suicidality data. Through a process of systematic review of literature according to PRISMA standards, screening and extracting studies with designs of a high level of evidence, and multi-level data synthesis, information is presented in the SURF database in a summarized, structured, and immediately relevant form. SURF allows users to search by risk factor, population, date, and indicator filters, enabling users to quickly access the most up to date and empirically grounded general or specific knowledge about suicide-related risk factors.

**Supplementary information:**

The online version contains supplementary material available at https://doi.org/10.1265/ehpm.24-00113.

Almost ten years after the World Health Organization’s recommendations for improved suicide prevention [1], suicide rates remain a major cause for concern, with no clear drop having been observed. Instead, the coronavirus disease 2019 epidemic has raised worries that mental health and socioeconomical consequences may translate into increased suicide rates [2]. In this context, the needs for reinforcing evidenced-based research and prevention efforts on the global scale have been stressed [3].

Clinical and epidemiological developments in suicidology have been mostly driven by the risk approach, which basically consists in identifying attributes, characteristics or exposures that raises one person’s odd of developing a suicidal outcome. On the individual level, professionals are required to integrate risk factors (RF) to estimate the likelihood of short-term suicidal behavior to deliver adapted protective and therapeutic assistance to suicidal individuals. On the populational level, RF also help non-governmental organizations and policy makers to identify populations with higher risk to tailor efficient selective prevention strategies.

For half a century, scholars have identified thousands of sociodemographic, clinical or biological predictors for suicide behaviours [4]. However, this large number of results still poorly translates into improvement of individual suicide predictions [4] and collective prevention [5].

As for any big data, the large number of results related to RF confronts scientists with the difficulty of accessing and synthesizing information to build original hypothesis or draw global inferences. To remove this barrier, we aimed to develop an interactive tool able to give the scientific community easy access to the highest and most recent evidence about suicide risk factor: the Suicide Risk-Factor – Data Query Tool (SURF-DQT – https://surf-dataquery.com/).

We designed the SURF as a web-application able to browse an extensive body of qualified results about suicide risk factors (the SURF database). To retrieve these results, we conduct an ongoing systematic review of the literature according to the PRISMA standards on the Medline database. The research algorithm is composed to identify all predictors of suicide, suicide attempt, suicide ideations and deliberate self-harm, while restricting the research to designs with presumed highest level of evidence (i.e. prospective cohorts, meta-analysis or times series – see Supplementary material).

Possibly relevant reports are screened for eligibility on title and abstract according to the following selection criteria: (a) historical or prospective longitudinal cohort studies, as defined by the STROBE criteria [6]; (b) studies evaluating the significance of one or several suicide-related clinical RF; (c) clear definition of the outcome as either suicidal behavior, suicide completion, suicide attempt, suicide ideation, deliberate self-harm, or suicidality; (d) English or French language. The last step consists of selecting articles to include from full-text analysis. Exclusion criteria are outcome poorly or unclearly defined; lacking numerical results; therapeutic trials; data mining methods or machine learning; focus on protective factors; non-suicidal self-injury as the sole outcome. Each search result is independently reviewed for eligibility by one author (MF). In the event of uncertainty, it is resolved by a second author (CEN).

The following standard data are systematically retrieved from included articles when available: first author, year of publication, country of first author, study design, DOI, PMID and web link. For each result extracted from the studies, we also retrieve the corresponding risk factor, outcome (i.e. suicide, suicide attempt, suicide ideations, self-harm, deliberate self-harm, suicidality, suicidal behaviour, suicidal thoughts, suicide intent, suicide plan, suicide risk), population, comparator, estimator, size effect and confidence interval or p-value. We only retain significant findings with available quantified data, the most adjusted results provided, and only results of prospective analysis methods. Data extraction is carried out by MF, with CEN providing validation and resolving any uncertainties.

To organize the thousands of results, we cluster the raw RF retrieved from papers by following a three-step analytical procedure inspired by content analysis of discourse (Strauss & Corbin, 1994): (a) normalization: we standardize formal variations and merge equivalent terms to get refined list of RF; (b) categorization: we group similar RF into categories that could parsimoniously encompass items based on the semantic similarity; (c) thematic integration: we infer broader, coherent themes based on RF categories. To ensure the accuracy of reported results, the raw RF terminology used in the original paper is also reported (original RF).

After an initialization of the application, we prolonged the first systematic review by an iterative process of searching, screening, extraction, and data synthesis to keep the database updated with the most recent publications. The database will continue to be updated on an ongoing basis according to this iterative process.

The web-app app consists of a Javascript frontend and backend with content management and data management features. It includes a custom structured data upload and processing pipeline, including checks for errors and a variety of edge cases, for administrators of the project. On the frontend it guides users through different touch points, allowing them to hone into the data they’re looking for incrementally. Users set their search criteria and then explore how multiple factors intersect using a matrix plot. Eventually users reach a tabular view that offers some background on matching studies that they can explore further, aiding them in their meta-analysis efforts.

The most recent search includes results from December 18^th^, 2013 until August 12^th^, 2022 [7]. The flow diagram is provided in Fig. 1. Of the included articles, there are 910 prospective/historical cohort studies and 114 meta-analyses.

**Fig. 1:**
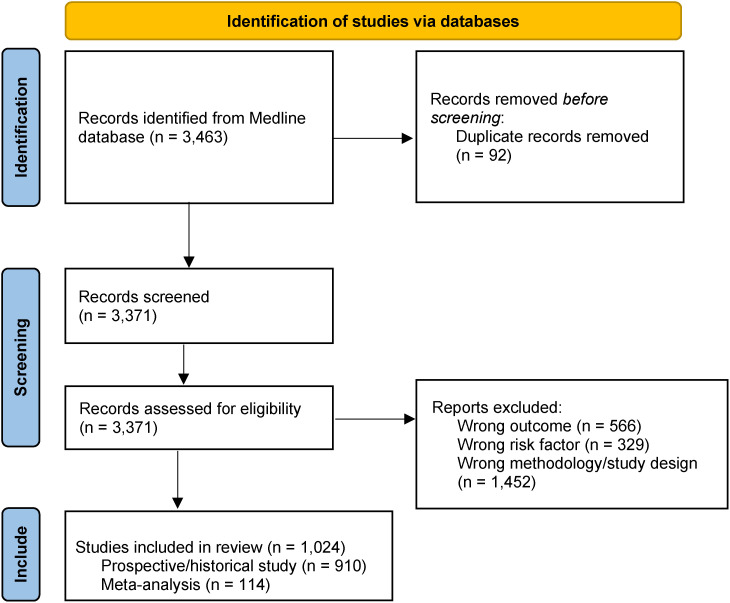
Flow diagram of the selection process used for the systematic review underpinning the SURF database.

The articles currently represented in the SURF database originate from 44 countries, with the top 5 countries including United States, Sweden, United Kingdom, Canada and China. The database contains a total of 9,911 results, detailing 40 RF categories (e.g. Race/Ethnicity/Language, Social Factors, Substance Use and Addictions, and Psychiatric and Mental Health Conditions) and 302 larger themes (e.g. Nationality, Social integration, Alcohol use and disorder, and Psychiatric treatment). Figure 2 presents a visualization of the number of available results, organized by RF categories and population groups.

**Fig. 2:**
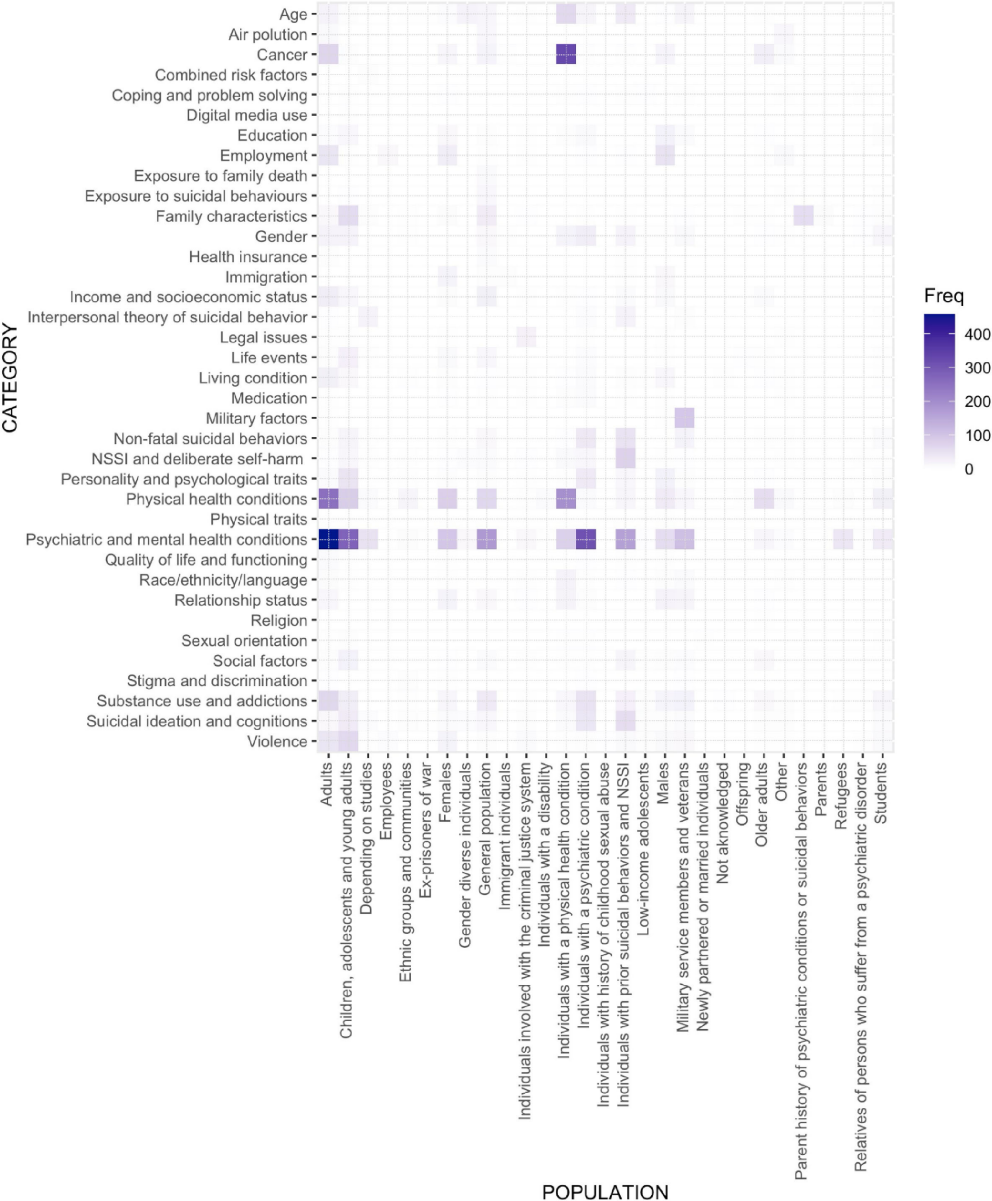
Heat map representing the magnitude of population and risk factor category values within our dataset.

The SURF allows users to search by RF, population, date, and indicator filters. Once a search has been input, results can be further filtered by year, estimator, and size effect. The search results are presented in the form of a graphical matrix that presents the outcome as intersections of the filters of interest (Fig. 3). Below the matrix, users can scroll a table in a standardized format that presents the outcome, population, RF, estimator, size effect, first author, year, country, article title, and article link for each individual result. App users are also provided with direct links to the included articles to ensure ease of access to the original publication.

**Fig. 3:**
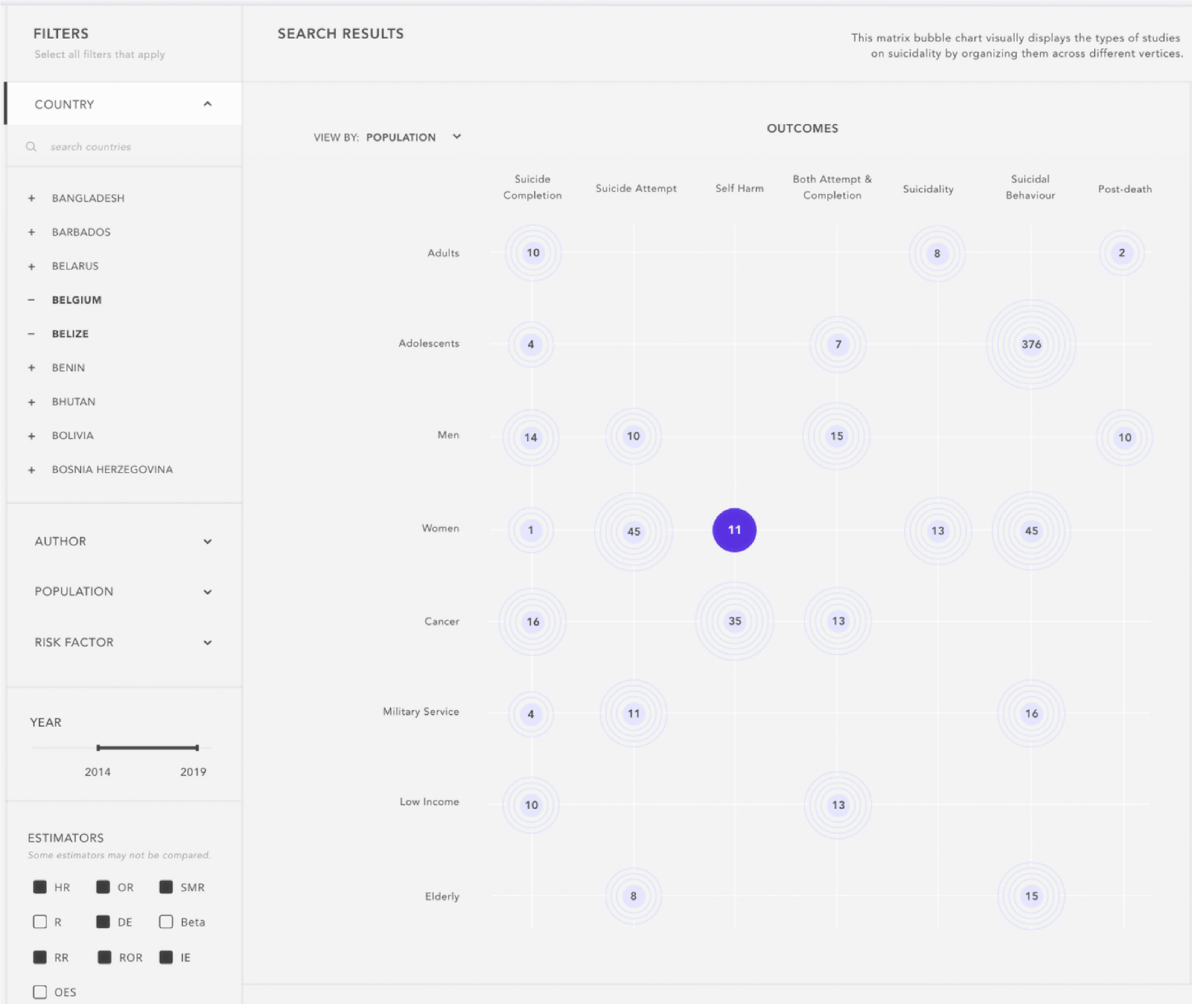
SURF application displaying the graphic matrix presenting the results of an example search.

The SURF web application is an interactive data synthesis and visualization tool that allows users to browse more than 9,911 high evidence results about suicide and suicidality. The underlying PRISMA-based systematic review and pre-filtering of studies based on the STROBE criteria ensures the exhaustivity and quality of these readily available results. By enabling the cross-combination of exploration criteria, it provides summarized, structured, and immediately relevant information as respect to the users’ interests.

The SURF could inform clinician’s practice by helping them easily find, and ultimately integrate, the risk associated with the characteristics or behaviors of the population they encounter. From this point of view, the SURF may support the evidenced-based approach of suicidality management by helping the integration of “individual clinical expertise with the best available external clinical evidence from systematic research” [8]. From a public health perspective, the SURF tool can also assist with the development of prevention strategies. The matching between needs and interventions is a crucial condition for developing efficient and targeted policies, which requires the identification of the most at-risk individuals [1]. Policy makers and experts therefore need the robust criteria provided by SURF to determine the relevant populations to target and tailor the deployment of actions to the characteristics of these populations. Finally, the SURF is intended to help researchers, students, and the community to quickly access the most up to date and empirically grounded general or specific knowledge about suicide-related risk factors.

The SURF has several limitations: (a) The algorithm a priori restricts the search to studies with designs of a high level of evidence to produce a manageable number of results. Because corresponding keywords are very general, it is unlikely that it couldn’t capture high-standard studies, which are recommended to explicitly state their design in their title, abstract or text; (b) The search is confined to the Medline database, where journals of most high-impact studies are indexed; (c) For manageability, we did not assess the quality of each included study. However, all references are readily accessible within SURF, allowing users to further refine their evaluations by reviewing the complete manuscripts.

The SURF tool meets an unmet need for an accessible, complete, synthesized, and ergonomic exploration of results about risk factors in suicidology from study designs with a high-level of evidence. The database of the SURF will require continuous updates. The SURF is likely to help clinicians, policy makers, researchers, and the broader community to translate and operationalize academic results into real-world solutions.

## Supplementary information

**Additional file 1:** Supplementary Material - Search Strategy.

## Declarations

### Availability of data and material

There are no new data associated with this article.

Database available at https://surf-dataquery.com/
