# Supplementary material for: The Suicide-Risk Factor – Data Query Tool (SURF-DQT): easy and handy access to the exhaustive base of highest evidence suicide risk factors
Source: Environ Health Prev Med. 2024 Dec 7;29:69. doi: 10.1265/ehpm.24-00113 (PMC11631554; doi:10.1265/ehpm.24-00113)
Supplement: Supplementary file 1 — Additional file 1: Supplementary Material - Search Strategy. [file ehpm-29-069-s001.doc]

Database: Ovid MEDLINE(R) ALL <1946 to October 30, 2020>

Search Strategy:

--------------------------------------------------------------------------------

exp Cohort Studies/

meta-analysis/

longitudinal*.mp.

cohort.mp.

prospective*.mp.

meta-analysis.mp.

1 or 2 or 3 or 4 or 5 or 6

suicide/ or suicidal ideation/ or suicide, attempted/

("suicid* behavio?r*" or "suicid* ideation*" or ("suicid*" adj "attempt*") or "self-harm" or "suicid* complet*" or "suicid* death").mp.

8 or 9

risk.ti,kf.

Risk Factors/ or Health Risk Behaviors/ or Risk/

predict*.mp.

"risk factor*".mp.

11 or 12 or 13 or 14

7 and 10 and 15

(20201031 OR 202011* or 202012* or 2021* OR 2022*).dt,ez,da.

***************************

Ovid MEDLINE(R) ALL <1946 to August 12, 2022>

1 exp Cohort Studies/ 2383031

2 meta-analysis/ 165590

3 longitudinal*.mp. 379252

4 cohort.mp. 824281

5 prospective*.mp. 1005091

6 meta-analysis.mp. 251582

7 1 or 2 or 3 or 4 or 5 or 6 3284688

8 suicide/ or suicidal ideation/ or suicide, attempted/ 65747

9 ("suicid* behavio?r*" or "suicid* ideation*" or ("suicid*" adj "attempt*") or "self-harm" or "suicid* complet*" or "suicid* death").mp. 50072

10 8 or 9 80486

11 risk.ti,kf. 615392

12 Risk Factors/ or Health Risk Behaviors/ or Risk/ 1053317

13 predict*.mp. 1994387

14 "risk factor*".mp. 1308660

15 11 or 12 or 13 or 14 3340294

16 7 and 10 and 15 6762

17 ("20201031" or 202011* or 202012* or 2021* or 2022*).dt,ez,da. 3588787

18 16 and 17 1359
